# Clinically available testing options resulting in diagnosis in post-exome clinic at one medical center

**DOI:** 10.3389/fgene.2022.887698

**Published:** 2022-07-22

**Authors:** Elizabeth K. Baker, Elizabeth A. Ulm, Alyce Belonis, Diana S. Brightman, Barbara E. Hallinan, Nancy D. Leslie, Alexander G. Miethke, Marissa Vawter-Lee, Yaning Wu, Loren D. M. Pena

**Affiliations:** ^1^ Division of Human Genetics, Cincinnati Children’s Hospital Medical Center, Cincinnati, OH, United States; ^2^ Department of Pediatrics, University of Cincinnati College of Medicine, Cincinnati, OH, United States; ^3^Division of Neurology, Cincinnati Children’s Hospital Medical Center, Cincinnati, OH, United States; ^4^ Division of Gastroenterology, Hepatology and Nutrition, Cincinnati Children’s Hospital Medical Center, Cincinnati, OH, United States

**Keywords:** exome sequencing, genome sequencing, post-exome clinic, sequencing reanalysis, undiagnosed disease

## Abstract

Exome sequencing (ES) became clinically available in 2011 and promised an agnostic, unbiased next-generation sequencing (NGS) platform for patients with symptoms believed to have a genetic etiology. The diagnostic yield of ES has been estimated to be between 25–40% and may be higher in specific clinical scenarios. Those who remain undiagnosed may have no molecular findings of interest on ES, variants of uncertain significance in genes that are linked to human disease, or variants of uncertain significance in candidate genes that are not definitively tied to human disease. Recent evidence suggests that a post-exome evaluation consisting of clinical re-phenotyping, functional studies of candidate variants in known genes, and variant reevaluation can lead to a diagnosis in 5–15% of additional cases. In this brief research study, we present our experience on post-exome evaluations in a cohort of patients who are believed to have a genetic etiology for their symptoms. We have reached a full or partial diagnosis in approximately 18% (6/33) of cases that have completed evaluations to date. We accomplished this by utilizing NGS-based methods that are available on a clinical basis. A sample of these cases highlights the utility of ES reanalysis with updated phenotyping allowing for the discovery of new genes, re-adjudication of known variants, incorporating updated phenotypic information, utilizing functional testing such as targeted RNA sequencing, and deploying other NGS-based testing methods such as gene panels and genome sequencing to reach a diagnosis.

## Introduction

Next-generation sequencing (NGS), as a method for massively parallel sequencing, has expanded the repertoire of diagnostic tools for constitutional disorders in genetics. Genomic technologies such as exome and genome sequencing (ES and GS, respectively) have been widely implemented in the genetics clinic since becoming clinically available in 2011 (exome sequencing) and ∼2018 (genome sequencing). Such comprehensive testing is useful to diagnose ultra-rare single-gene disorders, unusual presentations of known conditions, and disorders with limited phenotypic information. In addition, ES and GS have facilitated and accelerated new gene discoveries for human disease. In spite of the widespread clinical experience with ES since 2011, the diagnostic rate is estimated to be under 50% across indications. This diagnostic yield leaves at least half of those who had ES without a diagnosis. In general, the greatest yields occur in early and/or severe presentations, where the effect size of the gene in question is greatest (Stark et al., 2016) (Tan et al., 2017). Other scenarios with higher diagnostic yields include subcategories such as neurological disorders (Mergnac et al., 2021) (Trujillano et al., 2017) and multiple congenital anomalies (Farwell et al., 2015) (Quaio et al., 2020) (Retterer et al., 2016). Given the relatively low diagnostic yield of ES, there is ample room for the discovery of new approaches that utilize the NGS data from ES or GS.

There is a multitude of reasons why ES or GS may not be diagnostic. Some examples include an environmental etiology, lack of identified single-gene cause, a phenotypic expansion of a known condition, or failure to detect or identify a variant (Shashi et al.,. 2019). Emerging data support a role for post-exome evaluations, as new diagnoses can be made utilizing existing resources. These resources include deep phenotyping, consideration of a new or extended phenotype in known conditions, incorporation of updated traits into the analysis, reanalysis of the identified variants, and realignment of the exome data utilizing new genome builds or incorporating recently published data regarding new human disease genes. Several recent publications highlight the role of these evaluations in reaching a diagnosis, with an increase of 5%–15% of additional diagnoses (Bergant et al., 2018) (Nambot et al., 2018) (Baldridge et al., 2017) (Baker et al., 2019) (Eldomery et al., 2017) (Wright et al., 2018). We present our experience with iterative investigations in a post-exome clinic and the value of utilizing all of the available clinical tools to reach a diagnosis.

We present six patients from five families evaluated at our center that illustrate the use of additional testing options that resulted in achieving diagnoses. These patients had previous nondiagnostic assessments that included ES. We incorporated NGS-based methods such as GS, gene panels, and targeted RNA sequencing to assist with finding a diagnosis. All these tests are clinically available and should be considered in those who are not diagnosed but have a high suspicion of Mendelian etiology. We present our experience with various modes of clinically-available testing that facilitated new diagnoses.

## Methods

### Ethical compliance

All patients consented to clinical diagnostic testing per local hospital protocol. Verbal consent was obtained from all families for inclusion in the case series. This study was deemed to not represent human subject research by the Institutional Review Board at Cincinnati Children’s Hospital (IRB protocol 2022-0018).

### Participant selection

This study consisted of a retrospective chart review for six patients from five families who were evaluated at Cincinnati Children’s Hospital Medical Center (CCHMC) post-exome genetics clinic. The common characteristic for selection is that all cases had previous nondiagnostic exome sequencing, were believed to have a genomic cause and received a partial or complete diagnosis during a subsequent NGS-based test (exome reanalysis, a multi-gene panel, or GS). The evaluations were performed on a clinical basis.

### Genetic testing performed

The original nondiagnostic exome tests were completed between August 2013 and October 2019, and the subsequent diagnostic tests were completed between April 2020 and July 2021. All genetic testing was performed at commercial labs. Supplementary Table 1 discusses the ES metrics for each case.

## Results

### Cases

Patient 1 is a 14-year-old female who was evaluated due to unilateral right iris and chorioretinal coloboma, optic nerve anomaly, dystonia, spastic diplegia, global developmental delay, and dysmorphic facial features. She had extensive nondiagnostic genetic testing previously including comparative genomic hybridization microarray, ES trio, and mitochondrial genome testing (Supplementary Table S1). Ophthalmology had recently noted possible mild right microphthalmia as well as previous colobomas. On physical exam, she was noted to have spasticity, right eye iris coloboma, slight facial asymmetry, and wide palpebral fissures. Her ES trio had been performed almost 2 years prior to our evaluation (at 12 years old) and identified two variants of uncertain significance, one in *ABCB6,* NM_005689.3:c.2168G > A (p.Arg723Gln), and the other in *COL6A2*, NM_001849.3:c.2626C > T (p.Arg876Cys). Subsequent deletion/duplication analysis of *COL6A2* via array comparative genomic hybridization was negative. Given these nondiagnostic results, exome reanalysis was ordered. This revealed a heterozygous variant in *RARB*, NM_000965.4:c.844G > A (p.Gly282Ser), which was classified as likely pathogenic. This variant was absent from population databases and occurred *de novo* in the patient. The variant had previously been submitted to ClinVar with a likely pathogenic classification (Variation ID: 546932). This result supported a diagnosis of microphthalmia syndrome 12 (MIM# 615524).

Patient 2 is a 15-year-old male who presented to the genetics clinic for developmental delay, hypoventilation syndrome requiring BiPAP during sleep, intractable epilepsy, dyskinesias, static encephalopathy, spasticity, and cortical visual impairment. He had extensive nondiagnostic genetic testing previously, including epilepsy and seizure disorder gene panel, mitochondrial genome sequencing, and ES trio (Supplementary Table S1). Five years had passed since the original ES was performed (at 10 years old); therefore, exome reanalysis was ordered. This identified a heterozygous variant in *CACNA1E,* NM_001205293.1:c.1054G > A (p.Gly352Arg). This variant has been previously reported as a recurrent disease-causing *CACNA1E* change (Helbig et al., 2018). This result supported a diagnosis of developmental and epileptic encephalopathy 69 (MIM # 618285).

Patient 3 is an 11-year-old male with a past medical history of a neurodegenerative disorder of unknown etiology who was evaluated for increasing spasticity, progressive developmental regression, and ataxia. He had a brain and spine MRI revealing minimal Chiari I malformation with mild cerebral volume loss. He had broad genetic and metabolic testing that was nondiagnostic, including ES at 7-year-old (Supplementary Table S1). The only notable abnormal lab finding on his extensive testing with intermittently elevated lactates (highest lactate of 6.2). He continued to have neurodevelopmental decline with loss of speech, loss of mobility, loss of independent living skills, and increased spasticity. Due to his symptoms, mitochondrial disorders were high on the differential; therefore, the GS trio as well as the mitochondrial genome were ordered. GS revealed two variants in *VARS2,* in *trans* configuration*.* Although one of the two variants occurred *de novo*, the laboratory report stated that the phase was able to be determined from the sequencing data. The VARS2-related disorder (MIM # 615917) is a mitochondrial disorder that has phenotypic variability including progressive neurological regression and progressive spasticity. The first variant, which was maternally inherited, was classified as pathogenic, NM_001167734.1:c.1546G > T (p.Glu516*). This nonsense variant had been previously reported in the published literature in another patient with the VARS2-related disorder, with electron transport chain studies demonstrating Complex IV deficiency, a consistent finding for this condition (Bruni et al., 2018). The second variant, which occurred *de novo* on the paternally inherited allele, was classified as a variant of uncertain significance (VUS), NC_000006.12(VARS2_v003):c.1569+4A > G. This variant was absent from population databases and unreported in association with VARS2-related disorder. VUS was intronic but the in-silico analysis was inconclusive as to the potential effect on splicing; therefore, RNA studies were performed to assess whether the change in the DNA affects the RNA transcripts. This targeted RNA study demonstrated abnormal RNA splicing, allowing this variant to be upgraded by the lab from a VUS to a likely pathogenic variant.

Patient 4 is a 12-year-old male with developmental delay, ADHD, intellectual disability, and abnormal gait. Brain MRI performed at age 4 years showed prominent ventricles and cerebral sulci, along with incidental bilateral choroid plexus cysts. He was diagnosed with ADHD at 9 years. He has an intellectual disability, in the severely impaired range as defined by a full-scale intellectual quotient of 50 on the Wechsler Intelligence Scales for Children (fifth edition), and has an individualized education program (IEP). The family history includes a paternal history of an undiagnosed neuromuscular disorder and a brother with developmental delays. He had extensive nondiagnostic genetic work including karyotype, microarray, encephalopathy panel, and ES trio (Supplementary Table S1). ES trio performed with parents at 8 years old was nondiagnostic and identified one paternally inherited variant in *MICU1*, NM_006077.3:c.1A > G (p.?) This variant was classified as likely pathogenic and had been previously described in the published literature in the compound heterozygous state in a patient with MICU1-associated myopathy with extrapyramidal signs (O’Grady et al., 2016). This result was nondiagnostic and a second change within the *MICU1* gene was not identified. Subsequent exome reanalysis performed as a quad that additionally included his similarly affected brother identified a maternally inherited *SMC1A* variant, NM_006306.3:c.1903C > T (p.Arg635Cys). This variant had been previously reported in the setting of autism spectrum disorder (Kosmicki et al., 2017), and a different missense variant at the same amino acid residue had been previously described in association with Cornelia de Lange syndrome 2 (Huisman et al., 2017). This variant was classified as likely pathogenic by the laboratory and the result was consistent with a diagnosis of Cornelia de Lange syndrome type 2 (MIM # 300590).

Patient 5 (brother of patient 4) is a 9-year-old male with aortic coarctation status-post neonatal surgical repair, developmental delay, intellectual disability in the severely impaired range, and abnormal gait. Brain MRI performed at 1 year of age showed mild periventricular increased signal intensity with some mild white matter volume loss, thought to be most compatible with white matter gliosis from a prior injury, and bilateral choroid plexus cysts. A multi-gene autism/intellectual disability panel performed as a trio with parents at 9-year-old identified the aforementioned maternally inherited likely pathogenic *SMC1A* variant, NM_006306.3:c.1903C > T (p.Arg635Cys), consistent with a diagnosis of Cornelia de Lange syndrome type 2. The panel also incidentally detected two *MICU1* variants in the patient’s father, suggesting a diagnosis of autosomal recessive *MICU1*-associated myopathy with extrapyramidal signs. The aforementioned first variant, NM_006077.3:c.1A > G (p.?) in Patient 4, was classified as pathogenic by the clinical laboratory that performed this test, compared to the likely pathogenic classification assigned by the original laboratory. The second variant, NM_006077.3:c.937G > T (p. Glu313*), was classified as likely pathogenic. This nonsense variant was unreported in population cohorts, nor had it previously been described in association with disease. Concurrent exome reanalysis for the patient’s brother (patient 4), to which this patient’s sample was added for a quad case, identified that both brothers are carriers for their father’s likely pathogenic *MICU1* variant, NM_006077.3:c.1A > G (p.?) and share the maternally inherited *SMC1A* variant, NM_006306.3:c.1903C > T (p.Arg635Cys). The phase of the *MICU1* variants was not formally confirmed through testing for the father’s parents, but they are most likely in *trans* given that both patients 4 and 5 inherited only the c.1A > G (p.?) variant.

Patient 6 is a 14-year-old female with a past medical history of acute liver failure of undermined etiology status-post liver transplant. She was previously healthy 3 years prior to presentation when she had acute onset abdominal pain and nausea and was found to have profuse transaminitis, neutropenia, and hepatic encephalopathy consistent with acute liver failure. Extensive metabolic and genetic workup, including ES trio and mitochondrial studies, were nondiagnostic at age 12-year-old (Supplementary Table S1). One year after her liver transplant, she was noted to have neutropenia (absolute neutrophilic count ∼300) requiring Neupogen, pancreatic insufficiency requiring pancreatic replacement, and chronic kidney disease type II. This new phenotypic term was added for reanalysis 2 years later, which subsequently revealed a heterozygous change in *SPINK1*, NM_003122.4:c.101A > G (p.Asn34Ser). This variant was classified as a risk allele for autosomal dominant pancreatitis (MIM # 167800) and is a frequently reported variant in association with pancreatitis (Witt et al., 2000). Risk allele variants in *SPINK1* can have reduced penetrance, and the allele was paternally inherited. This result gives a partial diagnosis for her symptoms, specifically for her acute pancreatitis.

## Discussion

NGS provides a large throughput sequencing platform that has enabled many patients to be diagnosed with rare disorders through ES. In fact, recent ACMG guidelines endorse ES as a first-line test for multiple congenital anomalies and intellectual disability (Manickam et al., 2021). Despite this powerful tool, more than half of patients remain undiagnosed after ES. There are a number of possible reasons for a nondiagnostic ES result in patients for whom clinical suspicion for a genetic etiology is high. For example, a variant that is analytically detectable but in a candidate gene may either be categorized as having uncertain significance or remain unreported. In this setting, exome reanalysis may lead to a diagnosis if performed subsequent to the publication of a link between the disease and gene. Additionally, the clinical overlap between the features of the patient and the known phenotype of variants in the gene under consideration may contribute to the prioritization at the time of review. Finally, one must consider the limitations of NGS to detect clinically relevant variants. In particular, copy number variants, non-coding variants, and regions with poor sequencing coverage can be reasons for nondiagnostic results. In these instances, additional testing can lead to a diagnosis, as illustrated by some of the cases presented.

We illustrate several cases in which post-exome evaluations led to new genomic diagnoses. In our experience, the diagnostic yield of genomic sequencing can be enhanced by utilizing any and all of the following approaches: updates to the patient’s phenotypic data, reinvestigation of specific variants for pathogenicity with functional assays, reannotation of the existing genomic data, and consideration of dedicated testing such as single gene or panel assays that may maximize coverage. In selected cases that have out-of-date testing, updated testing with the latest technology may lead to a diagnosis. These tools are generally available clinically. We present this knowledge to increase awareness of the utility of ongoing evaluations in undiagnosed cases.

Diagnoses related to phenotypic expansion or recent description of relevance for human health.

Among our cases, diagnoses for patients 1 and 2 were reached due to the publication of new gene-disease associations between the completion of the patients’ exomes and their reanalysis.

Patient 1, who has chorioretinal coloboma, iris coloboma, optic nerve coloboma, dystonia, spastic diplegia, global developmental delay, and dysmorphic facial features has a *de novo* likely pathogenic variant in the *RARB* gene reported during exome reanalysis. *RARB* variants had been previously reported in association with autosomal recessive or autosomal dominant micro/anophthalmia, congenital diaphragmatic hernia, and varied additional clinical features that included spasticity and dystonia (Srour et al., 2013) (Srour et al., 2016). However, *RARB* had not been specifically related to colobomas until a subsequent study published after completion of patient 1’s exome more clearly linked heterozygous *RARB* variants to coloboma without microphthalmia, strengthening the link between this variant and the patient’s phenotype (Kalaskar et al., 2020). This case illustrates shortcomings in genotype-phenotype recognition, as ophthalmologic phenotypes such as coloboma and microphthalmia can represent a spectrum of findings.

Patient 2, who has hypoventilation syndrome, epilepsy, dyskinesias, static encephalopathy, spasticity, and cortical visual impairment, has a *de novo* pathogenic variant in the *CACNA1E* gene reported during exome reanalysis. The association between *CACNA1E* variants and developmental and epileptic encephalopathy was published subsequent to the completion of the patient’s original exome and reported on reanalysis (Helbig et al., 2018).

### Variants overlooked during analysis due to lack of phenotypic fit

Patients 4 and 5, two brothers, each had developmental delays, intellectual disability, and abnormal gait. Patient 5 additionally had a history of aortic coarctation. Exome reanalysis identified a hemizygous likely pathogenic variant in the *SMC1A* gene, consistent with Cornelia de Lange syndrome 2 (CdLS). This result explains their developmental delays and intellectual disability. This variant does not appear to explain their gait differences, and thus may represent only a partial diagnosis for them. This variant had not been highlighted during the original exome because it was felt to have insufficient overlap given that the reported clinical features did not include dysmorphic features and seizures, the two primary findings reported to be associated with CdLS type 2.

Patient 6 has a history of acute liver failure prompting a liver transplant. Exome reanalysis ordered after her later diagnosis of pancreatic insufficiency and chronic kidney disease identified a risk allele in *SPINK1*, providing an explanation for her pancreatic insufficiency. She was diagnosed with pancreatic insufficiency after the original exome was completed; thus, the *SPINK1* variant did not come to attention during the original analysis.

### Causative variants that were identified but not reported on initial exome analysis

As part of our quest to understand the variant identification and classification workflow, we asked the performing lab if a variant had been identified but not reported in the previous analysis. Patient 3 is an example. He has a history of spasticity, progressive developmental regression, and ataxia. A genome sequencing trio identified biallelic variants in *VARS2:* one classified as a pathogenic variant, NM_001167734.1:c.1546G > T (p.Glu516*), and one classified as a variant of uncertain significance (VUS), NC_000006.12(VARS2_v003):c.1569+4A > G. Subsequent informative RNA sequencing permitted reclassification of the VUS to likely pathogenic. Retrospectively, only the c.1546G > T variant had been detected by the lab that performed the original exome, but the patient’s submitted phenotype was considered to have insufficient overlap with the associated condition, particularly in the absence of a second detected variant, for this variant to be reportable, and the previous laboratory had not reported the c.1546G > T variant in *VARS2*.

## Conclusion

NGS has been instrumental in facilitating diagnoses in patients with rare disorders. However, the diagnostic yield has been surprisingly low. We illustrate multiple approaches for attaining a diagnosis in patients with a genetic etiology for their findings, which are widely available to clinicians. In cases that still remain undiagnosed, analysis on a research basis by taking into consideration candidate genes can lead to an additional diagnosis.

Furthermore approaches could include long-read sequencing, optical genome mapping, and integration of the RNA sequencing data with DNA sequencing. Long-read sequencing will allow for repeat expansions to be evaluated that may cause human disease as well as assess haplotype phasing (Liu et al., 2020) (Marwaha et al., 2022). Optical genome mapping will allow for structural variations to be detected including aspects that are not obtained on standard microarrays balanced translocation and orientation of microduplications and deletions (Mantere et al., 2021). RNA sequencing elucidates the effect of a DNA variant allowing for pathogenicity to be further assessed (Lee et al., 2020). Using these tools clinically increases the chance of a diagnosis (Marwaha et al., 2022). Other approaches can include research analysis pipelines, functional assays to assess the effect of candidate variants, and RNA sequencing.

## Data Availability

The original contributions presented in the study are included in the article/Supplementary Material, further inquiries can be directed to the corresponding author.
