# Supplementary material for: Clinically available testing options resulting in diagnosis in post-exome clinic at one medical center
Source: Front Genet. 2022 Jul 22;13:887698. doi: 10.3389/fgene.2022.887698 (PMC9355124; doi:10.3389/fgene.2022.887698)
Supplement: Supplementary file 1 [file Table1.DOCX]

| **Patient ID** | **Phenotype** | **Brain MRI and other neurologic studies** | **Previous Genetic Testing** | **Methods of Diagnosis Achieved** | **Exome Sequencing Metrics** | **Diagnosis** | **Why diagnosis made** | **Full or**  **partial diagnosis** |
| --- | --- | --- | --- | --- | --- | --- | --- | --- |
| 1 | 14-year-old female with right iris and chorioretinal coloboma, optic nerve anomaly, nystagmus, bilateral myopia, bilateral amblyopia, right eye exotropia, dystonia, spastic diplegia, global developmental delay, and dysmorphic facial features (mild plagiocephaly with facial asymmetry) | Brain MRI: Low-lying cerebellar tonsils with normal brain parenchyma and ventricular system | - - CGH microarray - - SNP microarray - - ES trio - - Mitochondrial genome testing | ES trio reanalysis | Exome sequencing was performed using the SureSelect CRE targeted capture method on the Illumina HiSeq 2500 sequencing system with coverage of 96.80% at 20X. Exome reanalysis was performed with the original raw binary base call (BCL) files passed through the lab’s present bioinformatics pipeline, with coverage of 97.25% at 20X. | Microphthalmia, syndromic 12 (MIM# 615524)  *de novo* heterozygous likely pathogenic variant in *RARB* (c.844G>A, Gly282Ser) | Phenotype update with microphthalmia, coloboma, and spasticity along with new case report describing the variable phenotype led to diagnosis | Full diagnosis |
| 2 | 14-year-old male with developmental delay, hypoventilation syndrome requiring BiPAP during sleep, intractable epilepsy (focal and generalized), dyskinesias, static encephalopathy, scoliosis, osteoporosis on bisphosphonates, spasticity, and cortical visual impairment | Brain MRI: Ventricles and extra-axial spaces are mildly prominent. The corpus callosum is small. | - - Epilepsy and seizure disorder gene panel - - Acylcarnitine profile, uric acid, carnitine level, lactate, and pyruvate - - Mitochondrial genome sequencing - - ES trio | ES trio reanalysis | Exome sequencing was performed using the NimbleGen V3 targeted capture method on the Illumina HiSeq 2500 sequencing system with coverage of >94.3% at 10X. Exome reanalysis was performed with the original raw binary base call (BCL) files passed through the lab’s present bioinformatics pipeline, with coverage of 94.83% at 20X | Developmental and epileptic encephalopathy 69 (MIM# 618285)  a *de novo* heterozygous pathogenic variant in *CACNA1E* (c.1054G>A, p.Gly352Arg) | This variant had been recently described three years after original ES in association with early onset epileptic encephalopathy | Full diagnosis |
| 3 | 11-year-old male with neurodegenerative disorder of unknown etiology who was evaluated for increasing spasticity, progressive developmental regression, and ataxia | Brain and spinal MRI: Minimal Chiari I malformation with mild cerebral volume loss and intermittent syringomelia at C5-C6 and T1-T6 | - - Karyotype - - Fragile X expansion testing - - SNP microarray - - Spastic paraplegia gene panel - - Angelman/ Prader-Willi methylation - –Mucopolysacc haridosis enzyme panel - - Urine organic acids, serum amino acids, very long chain fatty acids - - Purine and pyrimidine panel - - Creatine disorder panel - - ES trio | GS and RNA studies | Exome sequencing was performed using the SeqCap EZ VCRome 2.0 (Roche NimbleGen) or the IDT xGen Exome Research Panel V1.0 capture kit on the Illumina HiSeq 2500 sequencing system with coverage of 98.37% at 20X. Genome sequencing (GeneDx) was performed on an Illumina platform with coverage of 98.9% at 10X and a mean depth of coverage of 63X. | VARS2-related disorder (MIM # 615917)  GS revealed two variants in *VARS2*, in *trans* configuration, one classified as a pathogenic variant (c.1546 G>T, p.E516X) and one classified as a variant of uncertain significance (VUS) (c.1569+4A>G, IVS15+4A>G).  RNA sequencing demonstrated abnormal RNA splicing upgrading VUS to likely pathogenic | GS found intronic variant (c.1569+4A>G, IVS15+4A>G) that was classified as a VUS which affected splicing revealed by RNA sequencing. This RNA sequencing change the classification to likely pathogenic. The other variant (c.1546G>T, p.E516X) was not reported on ES since there was only variant noted and lack of phenotypic overlap | Full diagnosis |
| 4 | 12-year-old with developmental delay, ADHD, intellectual disability (IQ of 50), gait abnormalities (left flat foot with pronation at ankle and right pronounced arch with internal rotation at ankle, contractures, bilateral pes cavus, down slanting palpebral fissures, and camptodactyly at 4^th^ and 5^th^ digit | Brain MRI: prominent ventricles and cerebral sulci, along with incidental bilateral choroid plexus cysts.    EMG: “possible left peroneal injury versus atrophy of extensor digitorum brevis (EDB) | - - Karyotype - - SNP microarray - - Fragile X - - Multi-gene region of interest panel for neuromuscular diseases and encephalopathy - - ES trio | Exome reanalysis quad | Exome sequencing was performed using the SureSelect CRE targeted capture method on the Illumina HiSeq 2500 sequencing system with coverage of 94.88% at 20X. Exome reanalysis was performed with the original raw binary base call (BCL) files passed through the lab’s present bioinformatics pipeline, with coverage of 94.30% at 20X. | Cornelia de Lange syndrome type 2 (MIM # 300590)  maternally inherited likely pathogenic  *SMC1A* (c.1903C>T, p.Arg635Cys) | Re-phenotyping allowed this variant to be reported. Previously exome stated insufficient overlap given that the reported clinical features did not include dysmorphic features and seizures. | Partial diagnosis |
| 5 | 9-year-old male with aortic coarctation, PFO, developmental delay, intellectual disability, short bilateral halluces, hypertonia, and contractures | Brain MRI: mild periventricular increased signal intensity with some mild white matter volume loss, thought to be most compatible with white matter gliosis from prior injury, and bilateral choroid plexus cysts. | - - SNP microarray | Autism/ID panel | - | Cornelia de Lange syndrome type 2 (MIM # 300590)  maternally inherited likely pathogenic  *SMC1A* (c.1903C>T, p.Arg635Cys) | Autism/ID panel as an alternative approach from ES. | Partial diagnosis |
| 6 | 15-year-old with acute liver failure status-point liver transplant, neutropenia, pancreatic insufficiency, and CKD type II | Brain MRI: Patchy areas of increased T2/FLAIR signal abnormality are noted in the bilateral frontal lobes,  involving the deep white matter and periventricular white matter. | - - ES trio - - Mitochondrial studies | Exome reanalysis | Exome sequencing was performed using the SureSelect CRE targeted capture method on the Illumina HiSeq 2500 sequencing system with coverage of 96.99% at 20X. Exome reanalysis was performed with the original raw binary base call (BCL) files passed through the lab’s present bioinformatics pipeline, with coverage of 96.56% at 20X. Genome sequencing (GeneDx) was performed on an Illumina platform with coverage of 97.6% at 10X and a mean depth of coverage of 41X. | Autosomal dominant pancreatitis (MIM # 167800  Paternally inherited *SPINK1* (c.101A>G, p.Asn34Ser) | Re-phenotyping to include pancreatitis | Partial diagnosis |
